# Supplementary material for: Causal Relationship between Aging and Anorexia Nervosa: A White-Matter-Microstructure-Mediated Mendelian Randomization Analysis
Source: Biomedicines. 2024 Aug 16;12(8):1874. doi: 10.3390/biomedicines12081874 (PMC11351342; doi:10.3390/biomedicines12081874)
Supplement: Supplementary file 1 [file biomedicines-12-01874-s001.zip › Supplementary Figure/Figure S4/Singe SNP plot of MR analysis for TL to ubm-b-1604.pdf]

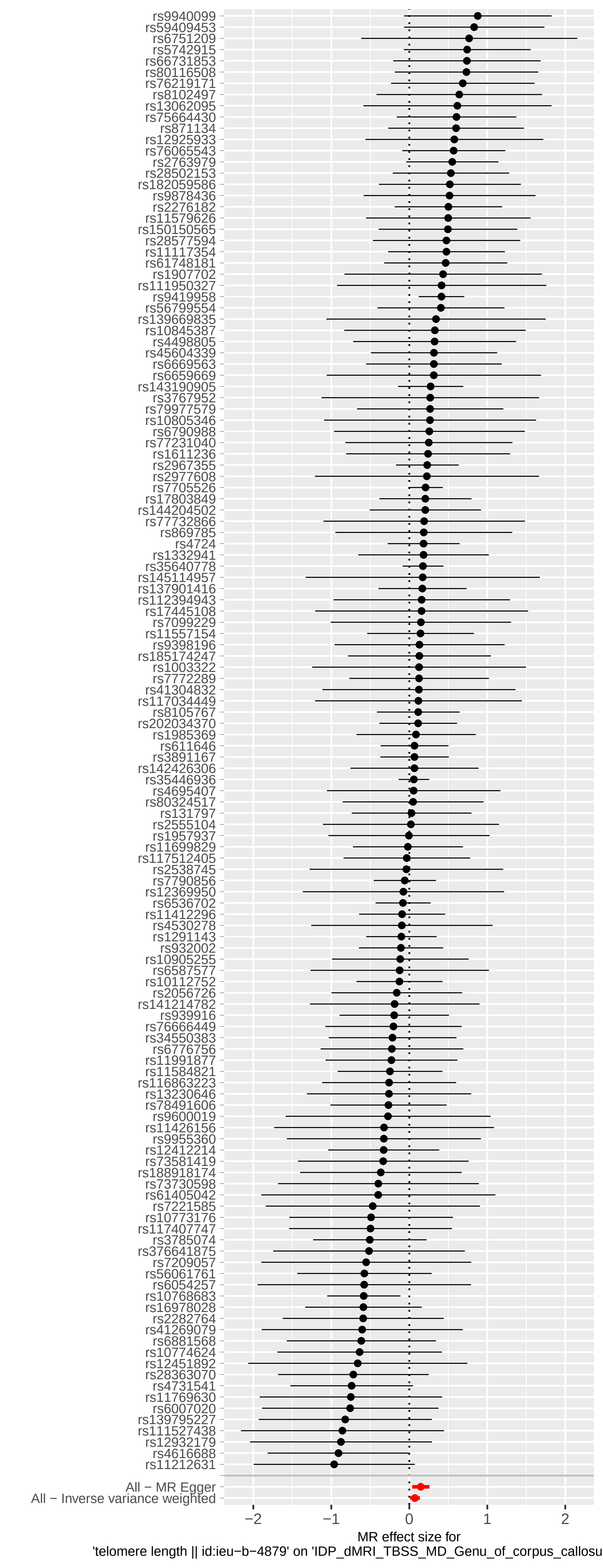
rs9940099
rs59409453
rs6751209
rs5742915
rs66731853
rs80116508
rs76219171
rs8102497
rs13062095
rs75664430
rs871134
rs12925933
rs76065543
rs2763979
rs28502153
rs182059586
rs9878436
rs2276182
rs11579626
rs150150565
rs28577594
rs11117354
rs61748181
rs1907702
rs111950327
rs9419958
rs56799554
rs139669835
rs10845387
rs4498805
rs45604339
rs6669563
rs6659669
rs143190905
rs3767952
rs79977579
rs10805346
rs6790988
rs77231040
rs1611236
rs2967355
rs2977608
rs7705526
rs17803849
rs144204502
rs77732866
rs869785
rs4724
rs1332941
rs35640778
rs145114957
rs137901416
rs112394943
rs17445108
rs7099229
rs11557154
rs9398196
rs185174247
rs1003322
rs7772289
rs41304832
rs117034449
rs8105767
rs202034370
rs1985369
rs611646
rs3891167
rs142426306
rs35446936
rs4695407
rs80324517
rs131797
rs2555104
rs1957937
rs11699829
rs117512405
rs2538745
rs7790856
rs12369950
rs6536702
rs11412296
rs4530278
rs1291143
rs932002
rs10905255
rs6587577
rs10112752
rs2056726
rs141214782
rs939916
rs76666449
rs34550383
rs6776756
rs11991877
rs11584821
rs116863223
rs13230646
rs78491606
rs9600019
rs11426156
rs9955360
rs12412214
rs73581419
rs188918174
rs73730598
rs61405062
rs7221585
rs10773176
rs117407747
rs3785074
rs376641875
rs7209057
rs56061761
rs6054257
rs10768683
rs16978028
rs2282764
rs41269079
rs6881568
rs10774624
rs12451892
rs28363070
rs4731541
rs11769630
rs6007020
rs139795227
rs111527438
rs12932179
rs4616688
rs11212631
All − MR Egger
All − Inverse variance weighted
−2
−1
0
1
2
MR effect size for
'telomere length || id:ieu−b−4879' on 'IDP_dMRI_TBSS_MD_Genu_of_corpus_callosu
